# Parsimonious data: How a single Facebook like predicts voting behavior in multiparty systems

**DOI:** 10.1371/journal.pone.0184562

**Published:** 2017-09-20

**Authors:** Jakob Bæk Kristensen, Thomas Albrechtsen, Emil Dahl-Nielsen, Michael Jensen, Magnus Skovrind, Tobias Bornakke

**Affiliations:** 1 School of Social and Political Sciences, University of Canterbury, Christchurch, New Zealand; 2 Department of Research, Nextwork A/S, Copenhagen, Denmark; 3 Department of Research, Analyse & Tal F.M.B.A, Copenhagen, Denmark; 4 Department of Sociology, University of Copenhagen, Copenhagen, Denmark; Universidad Nacional de Mar del Plata, ARGENTINA

## Abstract

This study shows how liking politicians’ public Facebook posts can be used as an accurate measure for predicting present-day voter intention in a multiparty system. We highlight that a few, but selective digital traces produce prediction accuracies that are on par or even greater than most current approaches based upon bigger and broader datasets. Combining the online and offline, we connect a subsample of surveyed respondents to their public Facebook activity and apply machine learning classifiers to explore the link between their political liking behaviour and actual voting intention. Through this work, we show that even a single selective Facebook like can reveal as much about political voter intention as hundreds of heterogeneous likes. Further, by including the entire political like history of the respondents, our model reaches prediction accuracies above previous multiparty studies (60–70%).

The main contribution of this paper is to show how public like-activity on Facebook allows political profiling of individual users in a multiparty system with accuracies above previous studies. Beside increased accuracies, the paper shows how such parsimonious measures allows us to generalize our findings to the entire population of a country and even across national borders, to other political multiparty systems. The approach in this study relies on data that are publicly available, and the simple setup we propose can with some limitations, be generalized to millions of users in other multiparty systems.

## Introduction

The representative opinion survey has long been the pinnacle of empirical research in political science [1,2]. The recent immense growth in digital platforms has provided researchers with the possibility of studying human behaviour on a whole new scale from traces left behind by our digital interactions [3]. From being limited to surveys with a couple of thousand respondents, political studies covering millions of people have emerged within the field of computational social science, generating important new knowledge about our digital and analogue lives.

Within the subfield of election forecasting, scholars have shown the potential for predicting election outcomes based on digital data from a diverse range of platforms including YouTube [4], Google [5], Twitter [6,7], Facebook [8,9], and even Wikipedia [10]. Studies based on the big social media platforms, that is, Facebook and Twitter, have largely been the most successful with prediction rates that in accuracy and scale often have outperformed traditional pooling [4] (see [11] for a general review). While this emerging field has mainly focused on predicting aggregated electoral results [12], a smaller group of studies has focused on the challenge of predicting individual political orientation [9,13–19]. Notably, Ceron et al. [11] were able to reach very high accuracies in their political profiling using only Twitter data, just as David et al. [18] displayed how political orientation can be determined by comparing individuals’ writing style with the writings on politicians’ public Facebook profiles. While many of these studies attain high prediction accuracies, this accuracy is often reached by limiting the study to the most active users [17]. Further, few studies validate their results against offline data such as surveys. These limitations have, however, been tackled in the work of Kosinski and colleagues who, in two papers ranked among the top 10 most influential papers in the history of PNAS (As calculated by Altmetric: https://pnas.altmetric.com/details/3058702 and https://pnas.altmetric.com/details/1294474), have shown how our personality and political attitudes can be predicted with great accuracy based solely on Facebook likes [13,14]. Applying machine learning algorithms to search for patterns in hundreds of diverse Facebook likes, these already famous experiments have thus disclosed how people’s preferences for Hallo Kitty and Harley Davidson can reveal details about their personality and political attitudes—often with better precision than their friends or family.

Thus far, the majority of studies predicting individual voting behaviour based on digital traces have focused on two-party systems or applied a left/right-wing scale, thereby avoiding the more challenging task of making all-inclusive predictions in multiparty settings, the principal political system of our time [18]. In this paper, we fill this gap by studying how individual party choice in a multiparty system is linked to liking posts made by political actors on Facebook. We base our prediction on likes for posts on public pages of Danish parties and politicians collected between January 2015 and 2017 through the Facebook Graph API. Through machine learning–based prediction models, we test how ‘political likes’, consisting of likes on posts created by politicians and parties, are able to predict present-day voter intention in a multiparty system for a subsample of surveyed respondents.

The main contribution of this paper is to show how public Facebook activity, even within a challenging multiparty system, can be effectively used to predict an individual’s present-day voter intention. Based on the simple measure of political likes, our models reach a prediction accuracy of between 60% and 70%, which are above any previous multiparty studies. Also, we show how even a single selective Facebook post-like can reveal as much about our present-day voter intention as hundreds of diverse likes drawn from our profile. In doing this we wish to challenge the current trend toward broader and bigger data running through the majority of studies within computational social science. By exploring the parsimonious measure of political likes, we make the point that though likes pertaining to everything from reality stars to soil types can accurately predict personal traits, more accurate and generalizable results in the case of present-day voter intention will tend to follow from a more parsimonious data strategy.

## Materials and methods

### Data

**Fig 1:**
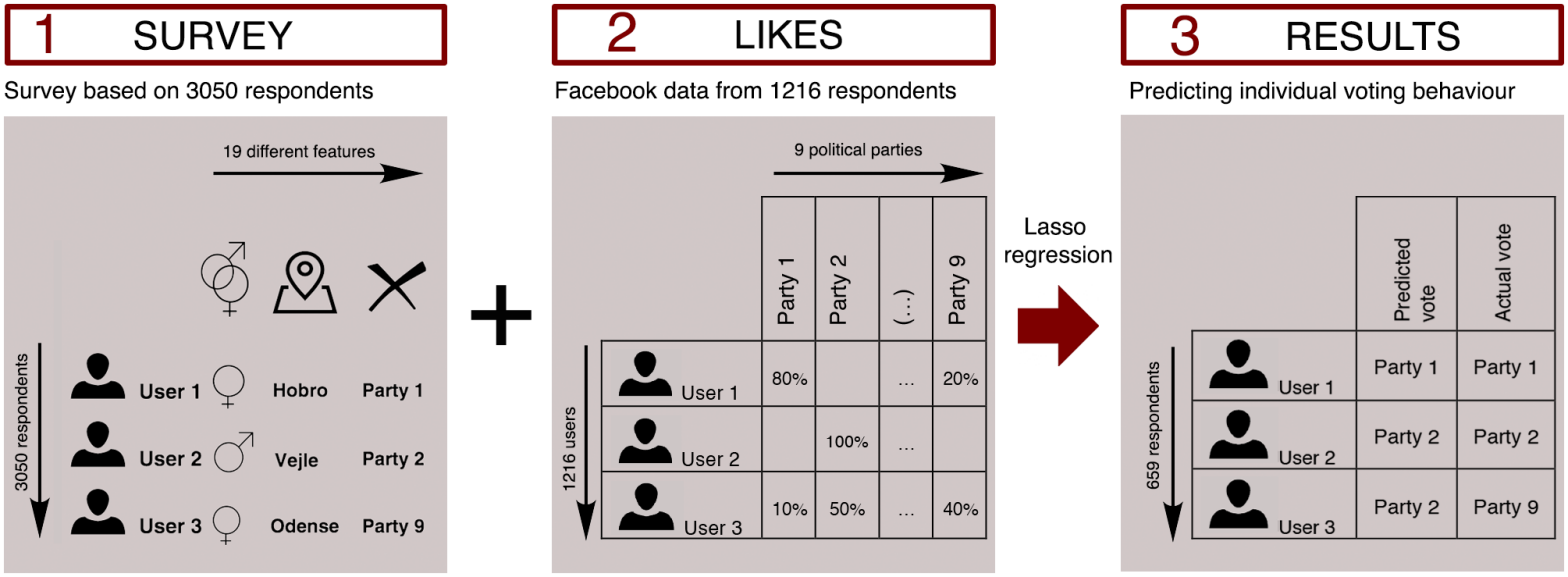
Data process. The process in three parts (1) A representative survey was completed by 3050 randomly selected people living in Denmark, providing information on standard sociodemographic qualities, political values, and present-day voter intention toward parties eligible in the general election. As shown in S3 Table, the sample is somewhat demographically representative of the country’s entire population. Respondents were subsequently asked to log in with their Facebook account, and if willing to accept the same, respondents’ public Facebook ID was stored. (2) Post-likes were independently collected from all public profiles of Danish parties and politicians on Facebook. (3) After completion of steps 1 and 2, we linked each respondent to the collected Facebook data and applied a LASSO-based multinomial logistic regression model to predict voter intention based on Facebook data.

We base our prediction on likes entered in page posts by Danish parties and politicians collected from January 2015 to 2017 through the public Facebook Graph API. Likes are a generic mechanism used by Facebook users to express their support of content that has already shown to be a good proxy for predicting both electoral results and personal traits [8,13,14], making it an immediate choice for exploring the possibility for predicting political orientation. For use in the final analysis, we only include respondents who were able, and willing, to share their public Facebook ID (N = 1216). We also limit our analysis to respondents who had liked political actors during the period and would vote for any of the nine parties currently in parliament (N = 659). The final sample is slightly smaller (~23%) than what we theoretically would expect given our database of political likes containing data from 1.3 million Danes. We ascribe most of this dropout to privacy concerns (S1 Table). As a result of this dropout, representativeness of the data sample becomes slightly distorted (see non-response analysis in S2 Appendix). However, for the most part, the distortion simply reproduces Facebook’s already skewed user groups with the only large bias being an underrepresentation of older users; a skew that was recently shown to have limited effect on how often a person would like political actors [20]. The data process is illustrated in Fig 1.

### Method

We test post-likes against a baseline model developed from survey data of sociodemographics, political values, and opinions on political issues developed based on current “best practice” within political science (see survey features in S5 Table). Moving on from the baseline model, we gradually compare a selection of multinomial logistic regression models, all predicting which party a person would vote for but modeled on different selections of Facebook data as well as combinations of Facebook and survey data. Using L1 regularization LASSO [21–23], only features that contribute significantly to the overall prediction are included in the models. In each model an L1 penalty was selected using 10-fold cross validation to avoid overfitting and account for variance in the prediction accuracy.

### Ethics statement

The survey was conducted by Userneeds, which is a professional European Marketing and Social Research company. Participants’ personal data is protected by Userneed’s privacy policy, which is in accordance with ESOMAR guidelines. Written consent was obtained specifically for this project and every participant was informed of the purpose of the study. Authors on this project only received fully anonymised data from Userneeds. The Human Ethics Committee at the University of Canterbury, New Zealand stated that formal committee approval was not necessary since researchers only had access to the anonymised data.

Data from Facebook used in this study was collected only from fully public repositories available through Facebook’s Public Graph API and in accordance with their Terms and Policies.

## Results

**Fig 2:**
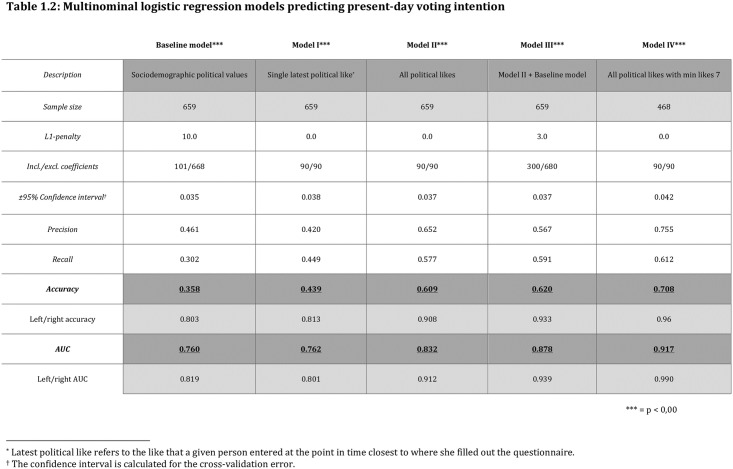
Multinominal logistic regression models predicting present-day voting intention.

The results depict how different uses of “political likes” are able to predict which of the nine parties in the Danish parliament a given person would vote for. The significance of the results is held against a null hypothesis that denotes no relationship between present-day voter intention and explanatory variables (*H*_0_: P = 1/9). The results are found in Fig 2.

### Establishing a baseline from sociodemographics, political values, and opinions

We initiate our analysis establishing a baseline model based on sociodemographic variables, political values, and opinions toward current issues collected through survey questions. The questions were selected to mirror the most typical variables for explaining voter alignment within the discipline of political science [24]. The baseline model (model 0) includes 19 different features. Note however, that several coefficients are neutralized by L1 regularization, which is implemented in LASSO regression to prevent model overfitting. The optimal model makes predictions with 35.8% accuracy (confidence interval [CI] of 2.9%) including 101 out of 668 coefficients. This echoes the accuracies of similar survey studies within political science, on average reaching an accuracy of approximately 35% (e.g. [24–26]). For comparison reasons, we calculate the same model’s accuracy for predicting present-day voting intention on a right versus left scale. Not surprisingly the accuracy is much higher when using this binary classification (80.3% accuracy).

### The power of a single political like

With an established baseline model, we turn toward our collected Facebook data. As an initial experiment, we create a model that uses just a single feature, the latest like that the respondent has entered to a post by a party or politician. This very simple setup (model 1) is more accurate and, on average, marginally better than our baseline model. With an accuracy of 43.9% (CI ±3.8%) and a right/left accuracy of 81.3%, model 1 indicates that a person’s single latest political post-like tends to say more about party choice than a prediction model trained on a sample with 19 different features on each person, including questions on core political values.

### Raising accuracy by including individuals’ entire political-like history

We now include all political likes for each person collected during the two-year period (model 2). The features in this model consist of the number of posts that a person has liked for each of the nine parties in parliament. For example, if a respondent has liked a post made by a party or a politician from that party on their public Facebook page, then that counts as one like to that party for that respondent. To compare respondents who are extremely active on public pages with those who are less active, all values are normalized across each respondent’s likes toward each of the nine parties. Applying these features, we predict which party a person would vote for with an accuracy of 60.9% (CI ± 3.7%). This result is notably better than both the baseline model and model 1. Interestingly, the best L1 penalty in model 2 was 0.0, meaning that excluding coefficients would not increase the cross-validated accuracy. With a right/left average accuracy climbing to 90.1%, the model suggests political likes as an efficient predictor for voter intention.

### Combining survey and political likes only minutely increases prediction rate

We now consider the possibility of a positive complementary effect by combining the best from two worlds. We add in the features of the baseline model to model 2 in order to explore whether the survey questions drawn from political science literature encapsulate other dimensions than the political likes: Do the two approaches overlap or complement each other? The new model, model 3, hereby includes all the sociodemographic background information, core political values from the baseline model, and the entire political-like history from model 2. The prediction accuracy is now 62.0% with (CI ± 3.7%). This is higher than model 2, but still within the margin of error. The increase in area under the (receiver operating characteristic, or ROC) curve (AUC) and in right/left accuracy, however, suggests that the model is still only slightly better than model 2.

The sample size in model 3 is lower than the number of coefficients, which is one probable explanation for why the added data does not deliver a significant increase in accuracy. Even though L1 regularization filters out most of the unnecessary noise, it is conceivable that the regression algorithm would perform much better with this selection of features if the sample size could correspondingly be raised.

### Optimizing political-likes prediction rates with minimum-like criteria

**Fig 3:**
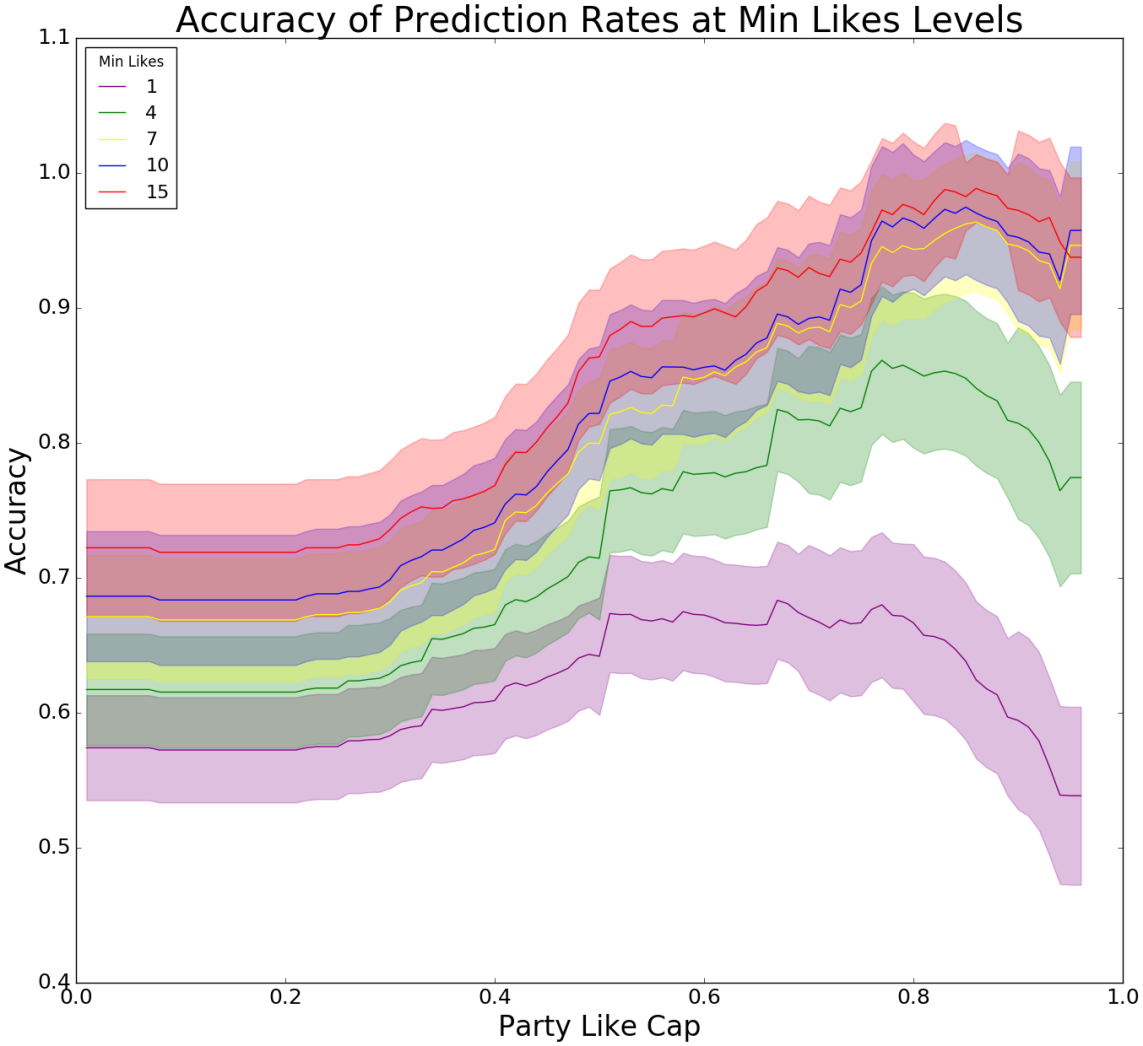
Accuracy at min likes levels. The x-axis shows party-like cap (PLC), which denotes how many likes in percentages that at the least go toward only a single party, meaning that at PLC = 0.8, only users who have at least 80% likes toward a single party are included. The y-axis shows the percentage of users who are accurately labeled. Each colored line shows accuracy for samples where all respondents have a minimum of total likes. Because the two criteria, party-like cap and minimum likes, involve filtering out respondents and thus effectively cutting down the sample size, it is unfeasible to rely on the training of machine learning algorithms for classification. Consequently, we made a simple algorithm that derives predictions based on the party a respondent has liked the most at different intersections of the two criteria.

The previous models propose political post-likes as the single strongest variable for predicting individual party choice. It is therefore reasonable to consider whether we can further optimize the use of this variable. Since we normalize the values for number of posts liked across each of the nine parties for each respondent, our models might make overconfident predictions based on respondents who have only liked a single political post. Similarly, a person for whom 90% of her likes go to the same party should yield better predictions than a person whose likes have been evenly distributed across four parties. We explore the relationship between these two criteria, namely (1) *minimum likes*, excluding respondents with less total likes than the threshold, and (2) *party like cap*, excluding respondents with a lower percentage of likes directed toward a single party than the threshold. The results can be seen in Fig 3 and Table 1 provides the values corresponding to the figure. Fig 3 shows how the accuracy increases with both min likes and party like cap indicating that respondents with many likes distributed to one or few parties yield the most accurate predictions. With, for example, min likes = 7 and party like cap = 0.8, prediction accuracy goes above 90%; however, sample size is down to 153, which also considerably increases the error rate (see Table 1).

**Table 1:** Prediction rates and sample sizes at different party-like caps with min likes = 7 (p < 0.001).

*Party-like cap*	0.0	0.5	0.7	0.8	0.9
*Sample size*	468	328	197	153	97
*95% Confidence interval (CI)*	0.046	0.05	0.058	0.059	0.062
*Accuracy*	0.64	0.777	0.861	0.912	0.93

### Thresholding total likes greatly increases accuracy

Most importantly, Fig 3 shows that overall prediction rates, when thresholding individuals on their total likes, begin to converge significantly with a total minimum of 7 political likes. Setting the minimum likes criterion higher than 7 results in only a little gain in total accuracy, but considerably reduces sample size. We therefore interpret a threshold of political likes at 7 as the best choice for a near optimal prediction rate.

Based on the optimization exploration, we deploy a fourth and final model that has the same features as model 2, but only includes respondents with a total of 7 or more likes for posts from parties or politicians. The effective sample size is now 468 while prediction accuracy has increased to 70.8% (CI = ±4.2%). It is indicative of better prediction rates by imposing a criterion for how many total political likes a user should have. Accuracy for right/left is now 96%.

## Discussion

**Fig 4:**
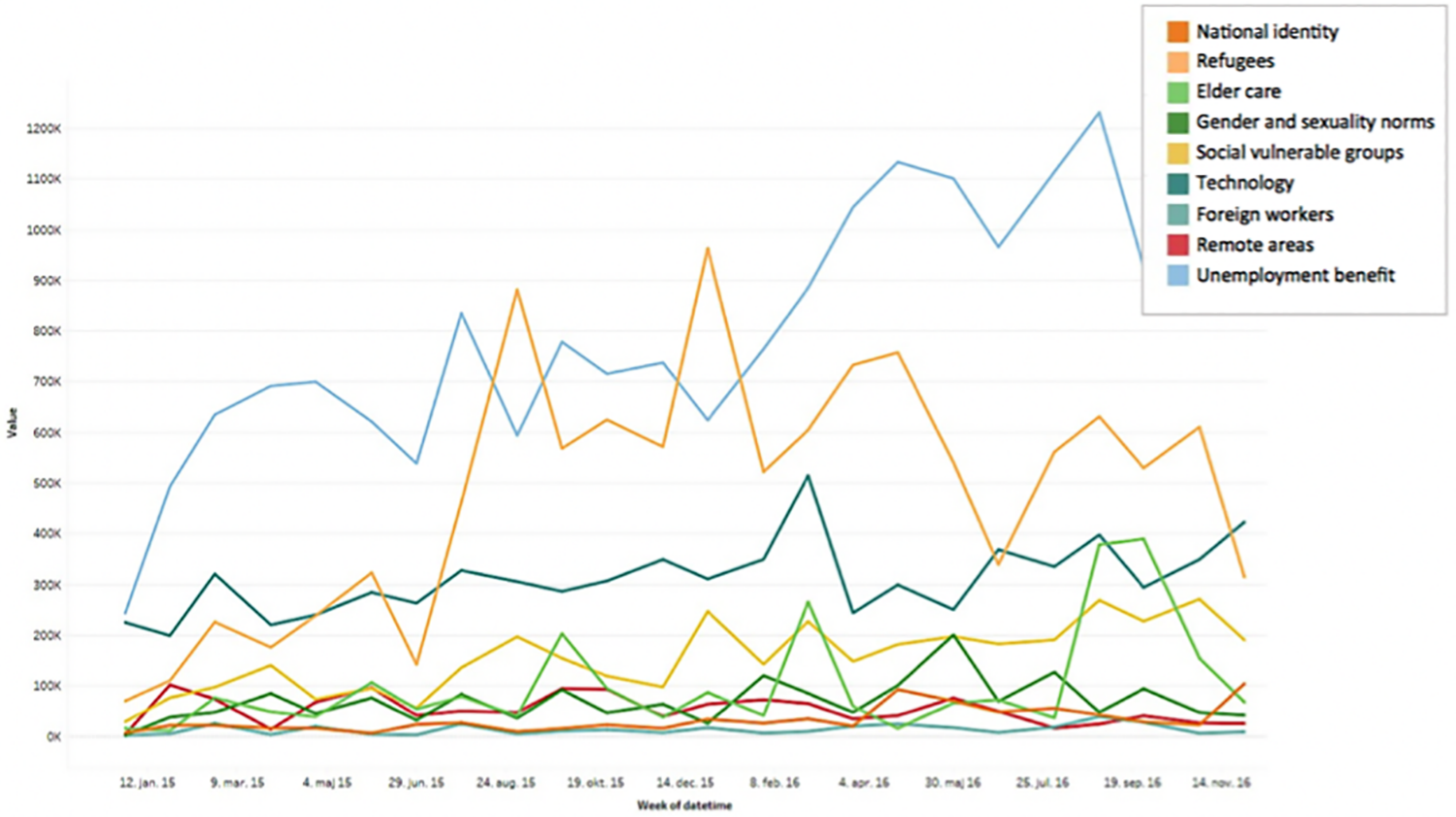
Combining trending news topics with users’ voter intention. Example showing trending topics for a group of users aligned with a specific party through their likes.

The main implication of our results is the potential for studying political behavior in multiparty systems on social media on a large scale and in near real time. The profiling of individual users through their political-like history thus lends itself as a tool to study political participation on social media. Through collecting political likes, we become able to profile approximately 1.3 million Danes—23% of the entire population—with a least one political like, and 1 million with at least seven. By filtering posts based on political segments defined by millions of likes, the approach offers scholars, practitioners, and politicians a view into democratic voters’ political dreams and the issues they engage in—all at a scale hitherto unknown to the discipline of political science. The approach could potentially add political significance to studies of trends and cluster formation in news consumption on Facebook as recently brought forth by Schmidt et al. [27]. Fig 4 constitutes a preeliminary experiment of studying a specific segment’s interest for particular political topics over time.

The technique is limited to the 23% of Danes who have liked political actors within the 2-year timeframe of the study. Due to the non-random subsampling in this study, which only considers respondents who are active Facebook users, the national population is not perfectly represented. Women, younger people and people with higher education are overrepresented in the samples used for the regression models (see S1 Appendix). However, we do not feel that any one group is totally left out or overrepresented to a degree that calls the overall results into question. Our own future studies, that is, predicting the aggregate electoral outcome based solely on political likes, however, are comparable to most opinion polls suggesting that our results can potentially be generalized to the entire Danish population (details in S5 Appendix). Further, tentative explorations of neighboring multiparty countries (Sweden, Germany, and Norway) show no indications that either the amount, or the usage of political likes should vary radically across national borders. Generalizing our findings outside the group of political likers, one should, however, be attentive of the bias inherent to Facebook as mentioned earlier. There are clear similarities between the our sample demographics and those found by Facebook’s own Audience Insights (https://www.facebook.com/ads/audience-insights/people?act=41292822&age=18-&country=DK).

Further, one should also expect political likers to be slightly more political active than the rest of the population [20]. With these limitations in mind, and in accordance with studies of other social media platforms [11], we estimate that the general mechanism of political likes would be reproducible in most open Western multiparty democracies where Facebook has become a central political arena.

### Toward parsimonious data

Searching for patterns within big datasets of diverse traces is not limited to Kosinski and colleagues’ work. Rather, the trend toward bigger and broader datasets seems to have become the standard for data experiments in the field of computational social science (e.g., [28]). Increasingly, this ideal has also appeared in commercial data analysis as illustrated by Cambridge Analytica, a data analytics firm drawing on Kosinsky et al.’s [13] work, when it proclaimed to have secured Donald Trump’s victory through the collection of “4–5,000 data points on every American” [29].

We share our colleagues’ fascination with the many stories about human behavior that these broad datasets can tell us. However, we argue that the field of computational social science by now has reached a level of maturity that makes it timely to replace the ideal of *broad* data with a parsimonious ideal of *selective* data. While the studies by Kosinski and colleagues should not be compared 1:1 (due to differences in goals and context), it seems fair to note how using only the respondents’ single latest political like delivers performance comparable to their best prediction of political attitude built on hundreds of likes [13,14] (AUC 0.8 (ours) vs. 0.85 (theirs)). [30] Also reach a result of AUC = 0.8 using the same dataset as [13]. Here we are reporting the left-right AUC of our study in order to make our results comparable. Future studies will reveal other areas to which parsimonious data strategy can be applied. Still, if our preference for “Hello Kitty” and “Harley Davidson” can accurately reveal our personal traits, then what greater expectations should we have of future predictions built on selective data linked to specific traits of interest?

### Why liking predicts voting: Contours of a theory

*Accuracy* with *generalizability* is the main advantage of our parsimonious data strategy. Based solely on this limited data scope, consisting of the single latest like per respondent, we were able to predict multiparty choice with an accuracy of 0.439. The accuracy was lifted above 0.6 by including all likes, and then above 0.7 by imposing a minimum like criteria of 7 likes. Our results thus indicate that even a single political like is comparable in accuracy to most multiparty studies in political science, commonly reaching accuracy of around 35%, by combining survey questions on sociodemographics, political values, and opinions toward current issues (e.g., [24–26]). While this line of research is not entirely comparable, with political scientists typically searching for explanation rather than prediction, the predictive power of political likes becomes striking when contemplating the approximately 30 survey questions involved in reaching 35% accuracy.

Given this background, it seems reasonable to consider why likes predict voting behavior dramatically better than survey questions: what makes a like predictive of our political behavior in the first place? Referencing major theories in studies of voting behavior, one could suggest that a like is predictive because it reveals alignment with the ideology of the liked party [1], the issue taken up [31,32], or the personal traits of the party candidate [33,34]. Our response to this question is to re-articulate an often-used designation: that likes comprise a generic mechanism for users to show their support. Political likes should be seen as a measure that captures a multitude of the abovementioned—and probably also other—theories for *why* we vote (i.e., ideology, shared issue, or personal identification). This response is in line with both the overall high accuracies reached, which makes it difficult to imagine a single theoretical driver, and with the lack of complementary effects seen in model 3, which suggest that we should view likes as encapsulating a number of different motives and preferences.

The high accuracies and lack of complementary effects also indicate that most people are highly selective with their political likes. We should thus not think of political likes as a cost-free interaction that we carelessly direct toward any post that catches our attention, but rather as an interaction form that we apply when we are clearly aligned across one or even multiple axes of preferences. As such, political likes should be seen as a parsimonious measure that condenses a heterogeneous mixture of different motives and individuals’ inscription into politics.

## Supporting information

S1 Appendix Data collection. (PDF)

S2 Appendix Non-response analysis. (PDF)

S3 Appendix Post-likes normalization. (PDF)

S4 Appendix Regression models. (PDF)

S5 Appendix Election prediction. (PDF)

S1 Table Data filtering and sample sizes. (PDF)

S2 Table Population and sample distributions for base demographics. (PDF)

S3 Table Population and sample distributions compared. (PDF)

S4 Table Results of non-response chi-squared permutation tests. (PDF)

S5 Table Baseline model: Predictive strength of individual features. (PDF)

S1 Fig Log of post-likes per user in main sample used in models (N = 659). (PDF)

S2 Fig Post-likes normalization procedure. (PDF)

S3 Fig Results of election prediction tests. (TIF)

S1 Dataset Anonymised survey responses and corresponding public Facebook actions. (ZIP)
