# Supplementary material for: Parsimonious data: How a single Facebook like predicts voting behavior in multiparty systems
Source: PLoS One. 2017 Sep 20;12(9):e0184562. doi: 10.1371/journal.pone.0184562 (PMC5607134; doi:10.1371/journal.pone.0184562)
Supplement: S1 Appendix — (PDF) [file pone.0184562.s001.pdf]

S1 Appendix. Data collection

Survey data

We collected data from a random sample of 3050 individuals via a European survey company. Variables corresponding to the questions posed in the survey can be found in Table S4. The survey was conducted in Danish, meaning all variable names are translated. At the beginning of the survey, respondents were given the option to connect their answers to their public Facebook profile. Interviewees were instructed that by agreeing to the terms we would get access to their public Facebook ID, but not any of their private content.

All data collected from interviewees used in this study were carried out with their full consent. In addition, each respondent retained the right to have her or his data deleted at any time.

Facebook data

All data were collected using the public Facebook Graph API 2.7[1]. The data cover the period from January 2015 and January 2017 and were harvested from a combination of media pages and political pages. The data were collected from 378 political pages covering politicians in parliament (or persons who have previously run for parliament) along with pages for political parties.

Combining Facebook and survey data

In order to use our two datasets (survey and Facebook), we had to create a sample cross-cutting both collections. To explore different filters and samples, we used a progressive filtering procedure, meaning that we kept adding filters gradually. The

[1] See https://developers.facebook.com/docs/graph-api/reference/v2.7/.

exploration is illustrated in Table S3. The filter represented in each row includes both the filter described and all previous filters.

We intend to compare all of our prediction models with the baseline model. Thus, we use the filtered sample (N = 659) in the case that the particular sample is overall easier to predict than the full survey for the baseline model. For models 1 – 4 we use the sample that includes all respondents with a political like. For model 5 we use only respondents with at least 7 likes (N = 468). Potential skews and biases as a product of the filtering process are covered in the non-response analysis.

We expect the demographic distribution for people who use Facebook to be different from that of the entire population of the country. Table S2 shows the distributions across demographics for which population values are publicly available; Table S3 shows the comparison between samples and population. From the comparison we can see that women, young people and people with higher education degrees are overrepresented in the n = 1216 sample, which consists of those who provided a public Facebook ID. In turn, men, older people and people with vocational training and primary school as their highest level of education are underrepresented. The distribution is relatively balanced for geographic categories. While the demographic distribution of the samples used in this study is not extremely skewed, it should still be kept in mind when interpreting the results. For a thorough analysis of all variables in the survey (including those for which population values are not available) see the non-response analysis in S3 and Table S4.
