# Supplementary material for: Parsimonious data: How a single Facebook like predicts voting behavior in multiparty systems
Source: PLoS One. 2017 Sep 20;12(9):e0184562. doi: 10.1371/journal.pone.0184562 (PMC5607134; doi:10.1371/journal.pone.0184562)
Supplement: S2 Appendix — (PDF) [file pone.0184562.s002.pdf]

S2 Appendix. Non-response analysis

In order to address the significant decrease in sample size from the full survey to the samples being used in our models, we have conducted a non-response analysis. The primary reduction in respondent numbers consists of individuals who did not have a Facebook profile or would not allow access to their public ID (N = 3050 – 1834 = 1216). Accounting for most of the dropouts are the 40% of the Danish population who do not have a Facebook account. We also expected privacy concerns to reduce participant numbers. The second dropout category is comprised of respondents who would not vote for one of the nine parties or did not enter at least one political like during the period (N = 1216 – 557 = 659). Accounting for the majority of these dropouts are people who did not plan to vote or who were not among the 28% (1.3 million) of Danes who liked political actors.

In order to determine the extent of skewedness for survey features, we did a 10,000-fold permutation test of chi-square (X^2) scores for each of the samples (see Table S4). The first test compares the distribution of answers from two random samples both from the full survey (total survey N = 3050). The next compares random samples where one is from the full survey and the other from a sample of only respondents with attached Facebook profiles (on Facebook N = 1216). The last compares random samples where one is from the full survey and the other from a sample of only respondents with at least one political like on any of the parties in parliament (with political like N = 659). The distribution of answers corresponding to a single feature, such as age, is only considered to be skewed if the mean of the X^2 value, resulting from the permutation test, lies outside of the 95% confidence interval of the first permutation test that compared two random samples from the full survey. For example, in the N = 1216 sample, gender has an X^2 mean of 0.96, but the 95% confidence interval of N = 3050 has X^2 values between 0 and 3.5, so the skew is not significant. However, the N = 659 has a gender X^2 mean of 4.49, and is therefore considered to have a small skew. Whether a skew is small or large is determined by the relative skew from the largest to the smallest. It is important to

remember that the degree of skew for a single feature is determined by its relation to *statistical* significance and not how skewed it is in the real world. For example, the most skewed feature, age, has a 10-percentage point difference between young and old.

Based on this exploration we see that the only large skew is age, as older users are underrepresented. A recent study in the neighboring country of Norway, however, found that age had very limited effect on how often a person would like political actors [24]. The same study also found that women and people with less education were more prominent in liking political actors. These results are in line with our dataset's smaller skews for these characteristics (see non-response analysis in Section S3). While the representativeness is thus within the expected bounds of a study on Facebook data, groups of people who do not act publicly, or are not on Facebook at all, are absent from the study [33].
