# Supplementary material for: Parsimonious data: How a single Facebook like predicts voting behavior in multiparty systems
Source: PLoS One. 2017 Sep 20;12(9):e0184562. doi: 10.1371/journal.pone.0184562 (PMC5607134; doi:10.1371/journal.pone.0184562)
Supplement: S3 Appendix — (PDF) [file pone.0184562.s003.pdf]

## S3 Appendix: Post-likes normalization

Post-likes are the simplest and most useful feature of all our Facebook features for predicting party choice. A vector represents each user with each field in the vector corresponding to the number of times that person has liked a post from a given party or politician from that party. As shown in Figure S1, number of post-likes per user has a big-head, long-tail distribution.

For this reason, the number of times that each user has liked posts from a party is divided by the total number of posts the user has liked. This effectively normalizes the sum of the vector for all users, which makes it possible to compare very active users with less active ones. An illustration of the creation of the normalized post-likes vector is shown in Figure S2.
