# Supplementary material for: Parsimonious data: How a single Facebook like predicts voting behavior in multiparty systems
Source: PLoS One. 2017 Sep 20;12(9):e0184562. doi: 10.1371/journal.pone.0184562 (PMC5607134; doi:10.1371/journal.pone.0184562)
Supplement: S4 Appendix — (PDF) [file pone.0184562.s004.pdf]

S4 Appendix. Regression models

All data analysis was carried out via Python. The code used for the regression models can be attributed mainly to Turi's GraphLab Create[1]; all other data analysis is original code. All data models are based on GraphLab Create's Logistic Regression module and implement only L1 regularization (L2 is set to 0). L1 regularization is used for selecting the coefficients corresponding to the features that deliver the best bias–variance tradeoff for generalizing the model. L1 regularization performs this selection by setting least important coefficients to exactly zero while decreasing other coefficients by a value relative to the chosen λ-value. The least important coefficients can roughly be defined as those least related to (least correlated with) the maximum log likelihood (MLE) of the model under a certain λ-value. The relation between the MLE and a given coefficient is determined by soft thresholding [34,35].

We report the following evaluative measures, all in the form of cross-validated averages:

> **AUC (area under [receiver operating characteristic, or ROC] curve):** Effectively states overall ability of the regression model for separating classes based on input variables with 0.5 denoting no relationship between explanatory variables and prediction rate. The thresholds for the ROC curve are incremented by 0.0001.
>
> **Precision:** Number of true positives out of all positives, or TP/(TP + FP). In multiclass cases, precision is calculated as the mean of all classes.
>
> **Recall:** Number of true positives out of all positives and false negatives, TP/(TP + FN). In multiclass cases, recall is calculated as the mean of all classes.

[1] See https://turi.com/products/create/docs/.

Accuracy: Global amount of correctly predicted classes divided by total sample size of the test data. In multiclass cases, this is not the mean of all class accuracies.
