# Supplementary material for: Parsimonious data: How a single Facebook like predicts voting behavior in multiparty systems
Source: PLoS One. 2017 Sep 20;12(9):e0184562. doi: 10.1371/journal.pone.0184562 (PMC5607134; doi:10.1371/journal.pone.0184562)
Supplement: S5 Appendix — (PDF) [file pone.0184562.s005.pdf]

S5 Appendix. Election prediction

To test the generalizability of our findings, we explored the ability for raw likes to predict the Danish general election in 2015 presented in Figure S3. Our initial attempt worked by counting each person as one vote toward the party that she liked the most. Through this approach, we obtained a mean absolute error (MAE) of 0.0271, not greatly different from the MAE of polls from the same period that averaged 0.010. Upon closer examination, however, it became apparent that the main cause of error was linked to overrepresentation of the Red-Green Alliance and underrepresentation of the Social Democrats. This is in line with the age skew in our data toward younger participants, as the Red-Greens are known for their large shares of younger voters where the Social Democrats are overrepresented among older voters.

Based on this premise, we developed an approximate weighting scheme that takes two random opinion polls produced at least a year before the election and uses them to create weights for the Facebook counts. The weight optimization process is a simple machine learning process that minimizes the residual sum of squares (RSS) between the Facebook count and the opinion polls chosen as the input. The Facebook count is made for the week leading up to the date of the opinion poll. Through this procedure, the MAE decreased to 0.011.
