# Supplementary material for: Parsimonious data: How a single Facebook like predicts voting behavior in multiparty systems
Source: PLoS One. 2017 Sep 20;12(9):e0184562. doi: 10.1371/journal.pone.0184562 (PMC5607134; doi:10.1371/journal.pone.0184562)
Supplement: S1 Table — (PDF) [file pone.0184562.s006.pdf]

S1 Table. Data filtering and sample sizes

Filter	Sample size
No filter, all respondents in survey	3050
Only respondents who reported their public Facebook ID in survey	1216
Only respondents who had liked at least one post on political pages corresponding to a party in parliament *(used in baseline and* ***models*** *II – III)*	659
Only respondents who had liked at least 7 posts on political pages *(used in model IV)*	468
