# Supplementary material for: Parsimonious data: How a single Facebook like predicts voting behavior in multiparty systems
Source: PLoS One. 2017 Sep 20;12(9):e0184562. doi: 10.1371/journal.pone.0184562 (PMC5607134; doi:10.1371/journal.pone.0184562)
Supplement: S2 Table — (PDF) [file pone.0184562.s007.pdf]

S2 Table. Population and sample distributions for base demographics[1]

Category	Population	n = 1216	n = 659
Female	0.5025	0.5444	0.5842
Male	0.4975	0.4556	0.4158
Age 18-34	0.2975	0.3497	0.3338
Age 35-53	0.358	0.3765	0.3849
Age 54-74	0.3445	0.2738	0.2813
Region Capital	0.3136	0.3519	0.3493
Region Central Jutland	0.2267	0.216	0.2287
Region Northern Jutland	0.1024	0.0984	0.0943
Region Zealand	0.1451	0.1005	0.1005
Region Southern Denmark	0.2122	0.2332	0.2272
Standard High School	0.1012	0.1278	0.1364
Vocational	0.3288	0.2393	0.2374
Ph.D	0.0066	0.0151	0.0101
Primary School	0.2861	0.1708	0.1724
Higher Education (2-4½ years)	0.1447	0.2194	0.2232
Higher Education (5 years or more)	0.0849	0.1661	0.1616
Higher Education (2 years or less)	0.0477	0.0616	0.0589

[1] Source for population percentages: http://danmarksstatistik.dk/da/Statistik
