# Supplementary material for: Parsimonious data: How a single Facebook like predicts voting behavior in multiparty systems
Source: PLoS One. 2017 Sep 20;12(9):e0184562. doi: 10.1371/journal.pone.0184562 (PMC5607134; doi:10.1371/journal.pone.0184562)
Supplement: S3 Table — (PDF) [file pone.0184562.s008.pdf]

S3 Table. Population and sample distributions compared

Category	n = 1216 compared to population	n = 659 compared to population
Female	0.0419	0.0818
Male	-0.0419	-0.0818
Age 18-34	0.0523	0.0364
Age 35-53	0.0185	0.0269
Age 54-74	-0.0707	-0.0632
Region Capital	0.0383	0.0357
Region Central Jutland	-0.0107	0.002
Region Northern Jutland	-0.004	-0.0081
Region Zealand	-0.0446	-0.0446
Region Southern Denmark	0.0209	0.015
Standard High School	0.0265	0.0351
Vocational	-0.0896	-0.0914
Ph.D	0.0085	0.0035
Primary School	-0.1153	-0.1137
Higher Education (2-4½ years)	0.0747	0.0786
Higher Education (5 years or more)	0.0812	0.0768
Higher Education (2 years or less)	0.0138	0.0112
