# Supplementary material for: Parsimonious data: How a single Facebook like predicts voting behavior in multiparty systems
Source: PLoS One. 2017 Sep 20;12(9):e0184562. doi: 10.1371/journal.pone.0184562 (PMC5607134; doi:10.1371/journal.pone.0184562)
Supplement: S4 Table — (PDF) [file pone.0184562.s009.pdf]

S4 Table. Results of non-response chi-squared permutation tests

	Total survey (N = 3050)			With Facebook ID (N= 1216)			With political likes (N = 659)			Degree of skew	
Category	X ² mean	0.025 quantile	0.975 quantile	X² mean	0.025 quantile	0.975 quantile	X² mean	0.025 quantile	0.975 quantile	N = 1216	N = 659
Party choice	8.08	3.11	15.23	17.42	9.81	27.67	19.91	9.92	29.24	Small	Small
Individual responsibility vs. Public responsibility	2.78	0.32	7.55	6.69	2.03	13.38	14.89	6.83	24.62	Not significant	Medium
Losing entitlement vs. Right to choose job	2.80	0.30	7.92	7.11	2.35	13.17	7.92	2.70	15.42	Not significant	Not significant
Social security reforms have become excessive vs. Just enough	2.80	0.38	7.51	3.38	0.67	7.61	6.08	1.86	12.94	Not significant	Not significant
Competition is healthy vs. Unhealthy	2.83	0.34	8.03	6.84	2.08	13.25	3.76	0.62	8.76	Not significant	Not significant
More freedom for corporations vs. Less freedom	2.87	0.31	7.85	1.95	0.23	5.05	5.90	1.53	12.61	Not significant	Not significant
People with high incomes do not pay enough taxes	2.81	0.34	7.33	4.21	0.85	9.20	7.00	2.06	14.19	Not significant	Not significant
Income inequality is too high	2.69	0.37	7.36	3.62	0.72	8.33	4.08	0.96	9.12	Not significant	Not significant
Violent criminals should face more severe punishment	2.79	0.32	7.37	1.89	0.22	5.17	2.63	0.38	7.23	Not significant	Not significant
More border control is desirable	2.85	0.38	7.80	6.97	2.21	13.87	7.83	2.50	15.09	Not significant	Not significant
We should do more to protect national heritage	2.82	0.33	7.81	5.81	1.61	11.62	6.63	2.12	13.67	Not significant	Not significant
We should prevent crime through counseling rather than punishment	2.79	0.38	7.79	6.06	1.71	12.27	6.84	2.02	13.55	Not significant	Not significant
Environment vs. Corporate growth	2.93	0.44	8.04	4.57	1.17	9.89	8.34	2.83	15.48	Not significant	Small
Homosexuals should have exactly the same rights as everyone else	2.71	0.32	7.70	7.95	2.84	14.19	8.32	2.94	15.62	Small	Small
Higher taxes on gasoline are desirable	2.91	0.37	7.82	9.44	3.80	17.21	7.38	2.46	14.30	Small	Not significant
Religious extremists have the right to freedom of public assembly	2.82	0.38	7.58	8.49	3.02	16.02	7.65	2.44	15.36	Small	Small
Gender	0.65	0	3.52	0.97	0	3.78	4.49	0.71	10.46	Not significant	Small
Age	1.33	0.03	4.59	27.09	16.69	39.90	14.59	6.75	25.87	Large	Medium
Geography	2.72	0.30	7.42	3.29	0.63	7.46	4.05	0.84	9.18	Not significant	Not significant
Education	5.53	1.46	12.54	16.98	6.62	28.92	24.74	14.84	36.18	Small	Small
