# Supplementary material for: Parsimonious data: How a single Facebook like predicts voting behavior in multiparty systems
Source: PLoS One. 2017 Sep 20;12(9):e0184562. doi: 10.1371/journal.pone.0184562 (PMC5607134; doi:10.1371/journal.pone.0184562)
Supplement: S5 Table — (PDF) [file pone.0184562.s010.pdf]

S5 Table. Baseline model: Predictive strength of individual features

**Category**	**AUC**	**Sample size**
**Gender****	0.617	659
**Age****	0.610	659
**Geography**	0.525	659
**Education****	0.624	659
**Individual responsibility vs. Public responsibility*****	0.712	659
**Losing entitlement vs. Right to choose job*****	0.653	659
**Social security reforms have become** excessive **vs. Just enough****	0.639	659
**Competition is healthy vs. Unhealthy*****	0.655	659
**More freedom** for corporations **vs. Less freedom*****	0.687	659
**People with high** incomes **do not pay enough** taxes***	0.700	659
**Income inequality is too high*****	0.686	659
**Violent** criminals **should** face more severe punishment	0.585	659
**More border control** is desirable***	0.687	659
**We should do more to protect national heritage*****	0.686	659
**We should prevent crime through** counseling **rather than punishment****	0.609	659
Environment **vs. Corporate** growth***	0.687	659
**Homosexuals should have exactly the same rights as everyone else**	0.497	659
**More** taxes **on gasoline** are desirable**	0.639	659
**Religious** extremists **have the right to** freedom of **public** assembly**	0.603	659

** = $p < 0.05$; *** = $p < 0.001$.S
