# Supplementary figures and images for: Parsimonious data: How a single Facebook like predicts voting behavior in multiparty systems
Source: PLoS One. 2017 Sep 20;12(9):e0184562. doi: 10.1371/journal.pone.0184562 (PMC5607134; doi:10.1371/journal.pone.0184562)

***S1 Fig. Log of post-likes per user in main sample used in models (N = 659)***

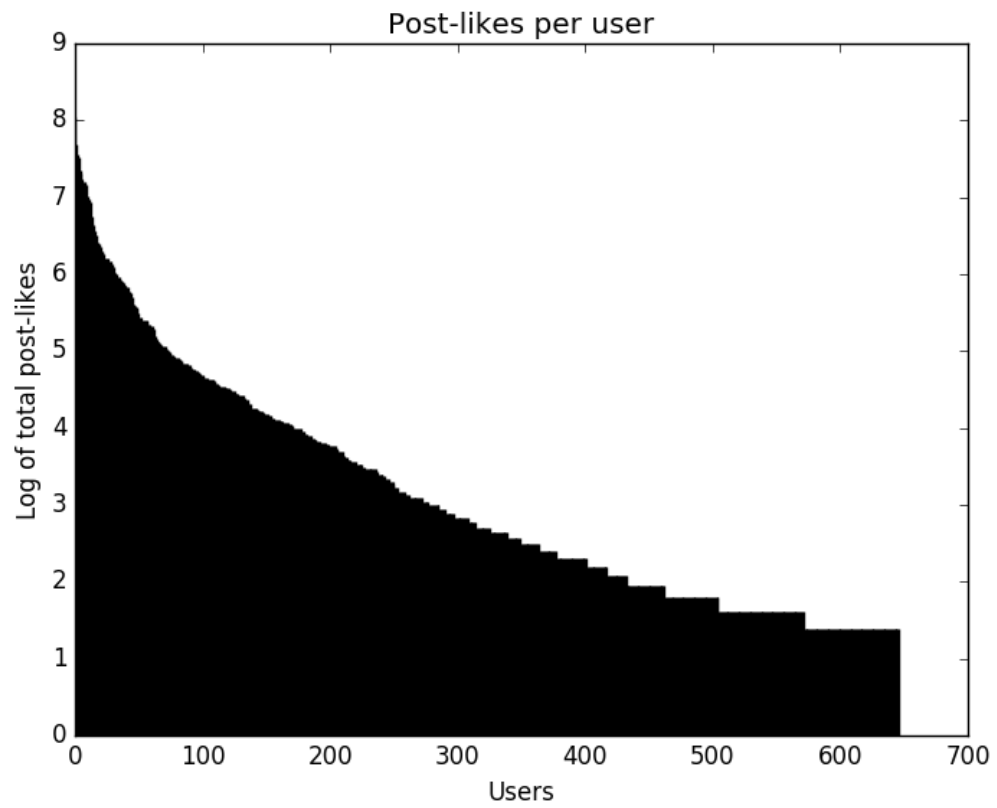

Supplement: S1 Fig — (PDF) [file pone.0184562.s011.pdf]

*S2 Fig. Post-likes normalization procedure*

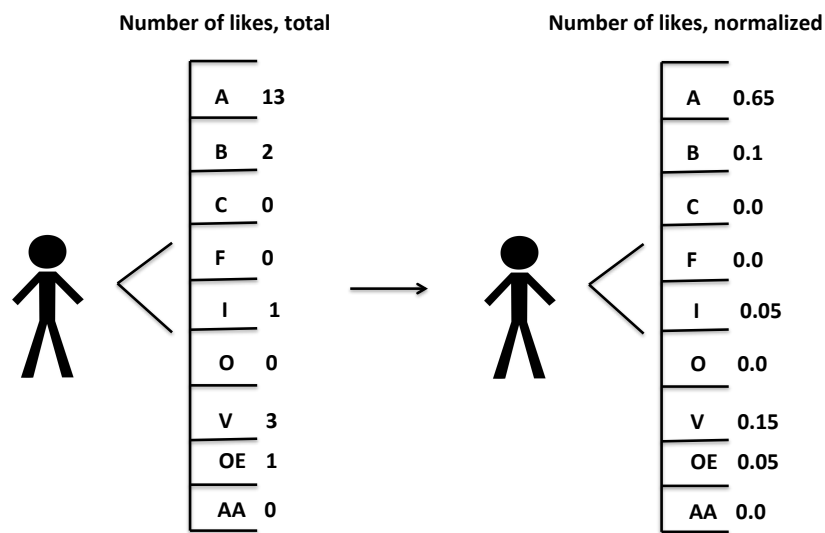

Supplement: S2 Fig — (PDF) [file pone.0184562.s012.pdf]

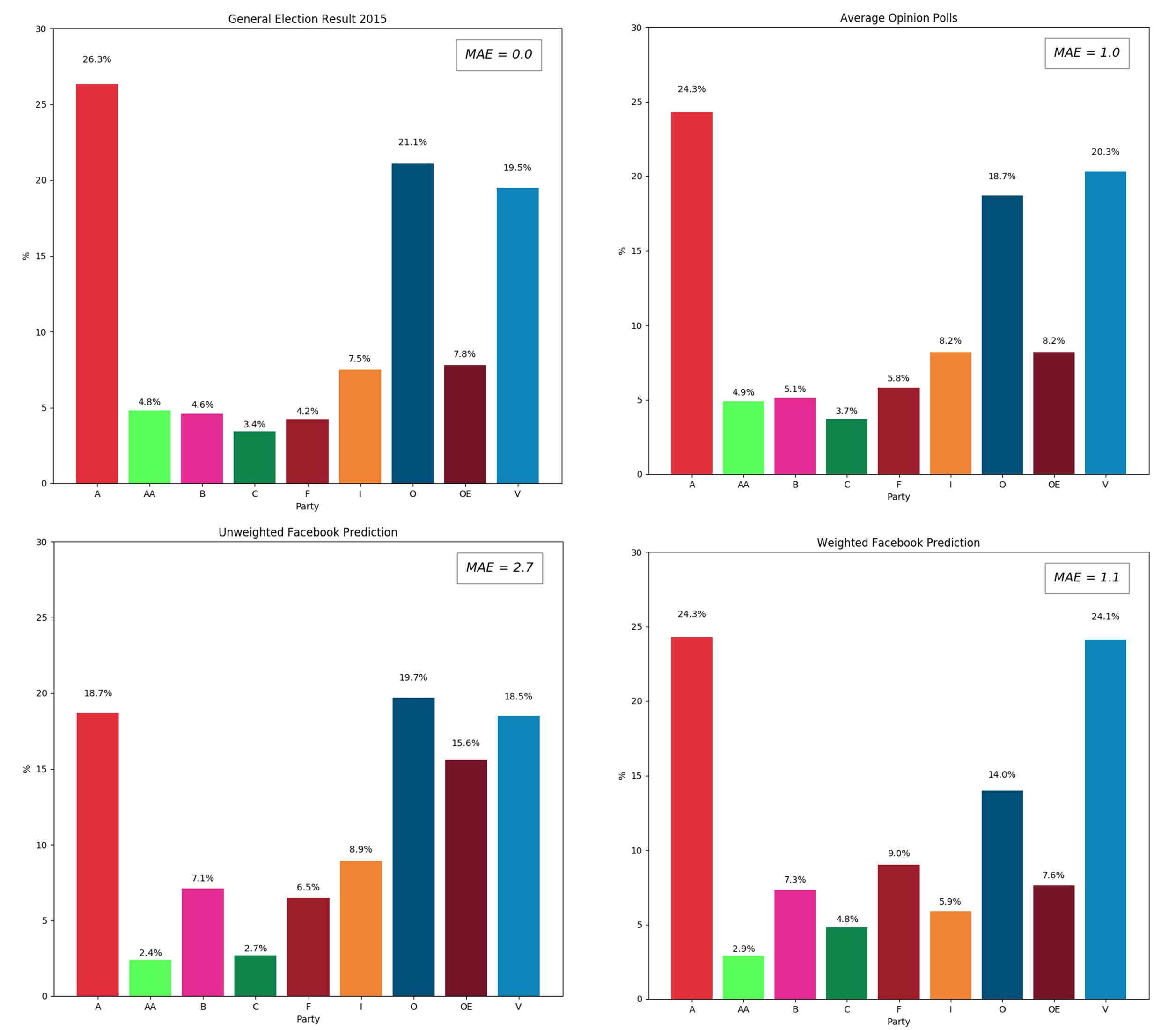

Supplement: S3 Fig — (TIF) [file pone.0184562.s013.tif]
